# Supplementary material for: An Effective Approach to Improve the Thermal Conductivity, Strength, and Stress Relaxation of Carbon Nanotubes/Epoxy Composites Based on Vitrimer Chemistry
Source: Int J Mol Sci. 2022 Aug 9;23(16):8833. doi: 10.3390/ijms23168833 (PMC9408316; doi:10.3390/ijms23168833)
Supplement: Supplementary file 1 [file ijms-23-08833-s001.zip › ijms-1823850-supplementary.pdf]

Supplementary Materials for

An effective approach to improve the stress relaxation and strength of carbon nanotubes/epoxy composites based on vitrimer chemistry

Yang Feng, Zhuguang Nie, Panhong Deng, Liping Luo, Xingman Hu, Jie Su, Haiming Li, Xiaodong Fan, Shuhua Qi*

Corresponding authors email address

Shuhua Qi*:qishuhua@nwpu.edu.cn

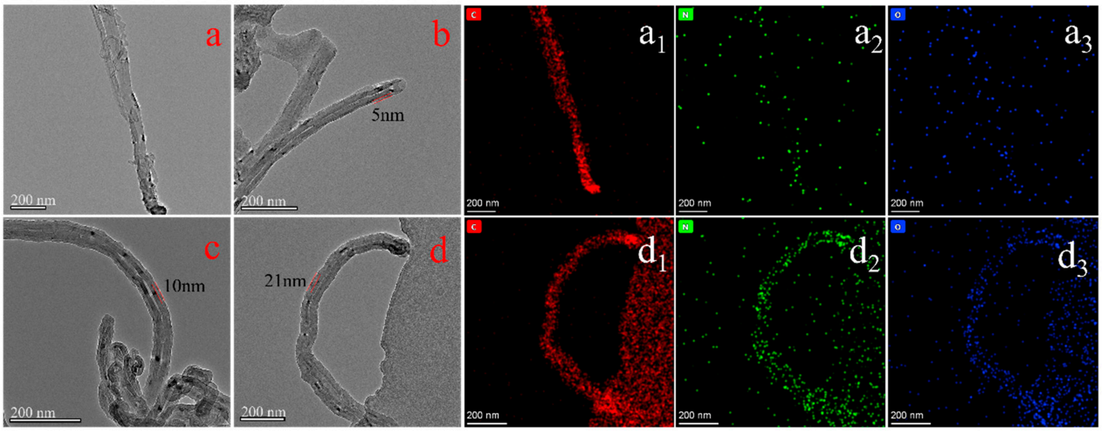

Figure S1. TEM images of pristine MWCNTs (a), PDA@MWCNTs with different PDA layer thickness (b, c, d). Elemental mapping images of MWCNTs and MWCNTs@PDA: (a_1, d_1) C. (a_2, d_2) N and (a_3, d_3) O.

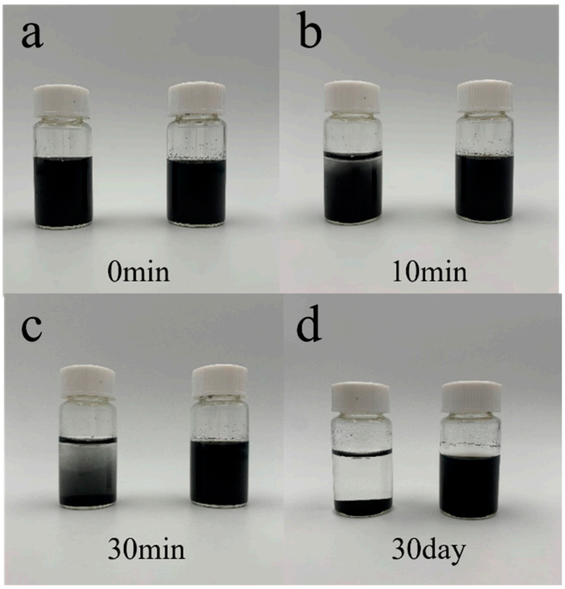

Figure S2. The photographs of MWCNTs (left) and MWCNTs@PDA (right) dispersed in water at (a) 0min, (b) 10min, (c) 30min, (d) 30days.

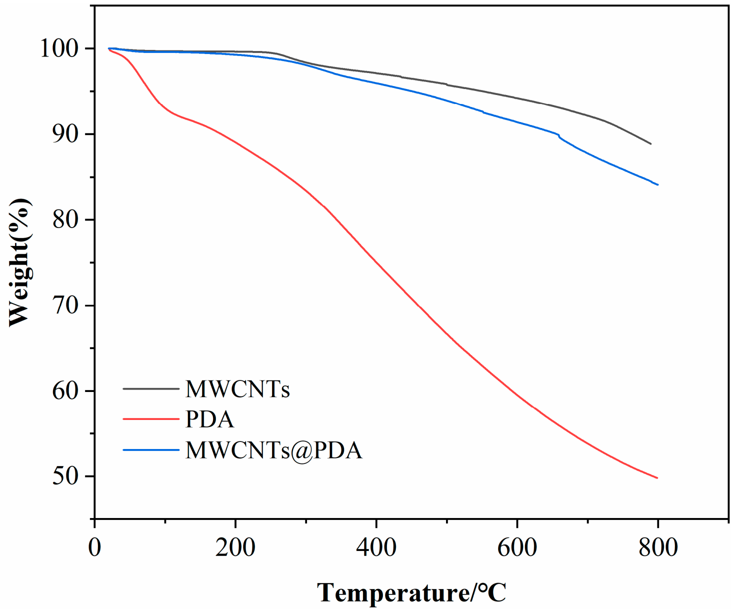

Figure S3. Thermogravimetric analysis of the MWCNTs, PDA, and MWCNTs@PDA
